# “It Can Be Quite Daunting”: Promoting Mental Health Service Use for Vulnerable Young People

**DOI:** 10.3390/healthcare13141740

**Published:** 2025-07-18

**Authors:** Anne Gu, Michelle Kehoe, Kirsty Pope, Liza Hopkins

**Affiliations:** 1 Department of Occupational Therapy, Monash University, Frankston 3199, Australia; annegu1400@gmail.com (A.G.); kirsty.pope@monash.edu (K.P.); 2 Alfred Health, Moorabbin 3189, Australia; l.hopkins@alfred.org.au

**Keywords:** youth mental health, volunteers, models of care, vulnerability, service delivery, qualitative research

## Abstract

**Background**: Today, young people face a variety of social, environmental and psychological challenges, making them more vulnerable to developing mental health issues. Worldwide 15% of adolescents experience poor mental health, with the majority not seeking help or receiving care. Therefore, it is critical that youth mental health services become more youth-friendly to encourage help-seeking. This study examines a new pilot volunteer model of care introduced into a youth mental health service in Melbourne, Australia. The aim of the study is to explore staff perspectives of the volunteer model. **Methods**: A qualitative research design was undertaken using semi-structured one-on-one interviews. Eight staff participated. Data was thematically analysed using an inductive approach. **Results**: Two main themes, ‘promoting service use’ and ‘implementation to practice’, were generated, along with sub-themes. The themes highlight benefits to staff such as reductions in workload and benefits to volunteers through the gaining of experience and knowledge. However, there was a need to support volunteers through greater training and supervision. **Conclusions**: Volunteers in youth mental health services can create a welcoming environment which enhances access and engagement for young people seeking help. Volunteers in a youth mental health setting can enhance accessibility, reducing staff workload and fostering meaningful engagement.

## 1. Introduction

Mental ill health is arguably among the most pressing of youth health issues worldwide [1]. Globally it is estimated that 15% of 10- to 19-year-olds experience mental health conditions, with suicide being the third leading cause of death among those aged 15 to 29 years [1]. Despite these alarming statistics, many mental health conditions in young people remain unrecognised and untreated [1]. Similar rates are reported in Australia, where 40% of young people aged 16–24 years have experienced a mental health issue in the preceding 12 months [2]. Mental health issues are estimated to cost the Australian economy up to $220 billion annually due to lost productivity and other direct and indirect costs [3]. As such, the implications for young people, their families, communities and the economy are immeasurable.

More than 75% of mental health issues emerge in young people before the age of 26 years when there is rapid physical, sexual, social and emotional change [4]. Following onset, mental health can persist into adulthood, impacting the young person’s capacity to fulfil their potential [5]. This vulnerability can limit access to health care, impact education and occupation outcomes and expose the sufferer to stigma, discrimination and social isolation [6]. As such early intervention, prevention and help-seeking strategies are key aspects of addressing vulnerable young people’s mental health concerns. However, in the past, many traditional Australian mental health services were critiqued for not being youth-friendly, thereby reducing help-seeking by those in need [7].

### 1.1. Volunteers in Health Services

In many cases, health service volunteers act as conduits between vulnerable patients and their families, and health care staff [8]. Despite the inclusion of volunteers more broadly across the health system and within society, reports on the use of volunteers in the mental health system are scant, even more so within youth mental health. The benefits to the volunteer and the person interacting with the volunteer, in a more general health setting, have been widely reported and include improved trust, the sharing of information and social interaction [8,9,10]. In addition to the benefits volunteers bring to service users, the dynamics between staff and volunteers are critical to the success of such programmes since both volunteers and mental health staff play supportive roles within their own capacity [11]. The literature suggests that, while volunteers can alleviate staff workload and enhance service delivery, the integration of volunteers into existing teams can also present relational challenges. These include role ambiguity, communication gaps and differing expectations between paid staff and volunteers [12,13]. However, staff have also identified challenges in working with volunteers such as volunteer training needs, coordination and role and general management, which can take time [8,14]. For example, there is often a need to coordinate volunteer activities to align with patient demand and daily routines [14]. Further issues are the transient nature of the volunteer role, which is not employment, and the need to ensure the volunteer role is cultivated through training and supervision to ensure greater longevity [14]. However, for volunteers the additional time needed for training and supervision can act as a barrier to committing to the role [14]. Although the concept of time is a common theme in the volunteering literature, there is scant evidence on how services manage the downtime of volunteers, highlighting a lack of research in this area.

Positive staff–volunteer relationships may be fostered through clear role definitions, mutual respect and structured supervision [15]. However, when these elements are lacking, tensions may arise, potentially undermining team cohesion and service quality. As such, from a staff perspective, there appears to be a fine balance between the benefits of having volunteers to support patients and the challenges of staff supporting volunteers. To date, there has been limited research which examines the relationship between paid staff and volunteers within a mental health setting; however, one notable study by Pinto De Costa and Oliveria [11] concluded that staff felt there were significant opportunities to impact both volunteers and patients, but greater support and training was needed to support the volunteers in order to develop an effective service.

### 1.2. Theoretical Overview

The role of volunteers can vary across different models of service delivery across the world [12]; however, volunteering generally refers to an individual providing their unpaid time to benefit others [13]. Volunteers in mental health services tend to occupy a unique position that distinguishes them from both clinical staff and clients. They often act as a relational bridge, providing informal, peer-like support that can ease the transition into care, particularly for young people who may be apprehensive about engaging with formal mental health services. This bridging role highlights how volunteers can reduce stigma, foster trust and enhance client engagement through their non-clinical, approachable presence [16,17]. However, most studies in this space have focused on this relationship between the volunteer and the client, with relatively less attention paid to the relationship between the volunteer and the broader service, including staff such as administration officers [16].

Unlike mental health professionals, volunteers are perceived as neutral and relatable, which can facilitate open communication and emotional comfort for the client, whilst enabling staff to make the time to support their patient [16]. This dual impact, supporting both service delivery and client experience, underscores the importance of understanding volunteer roles not just as auxiliary, but as integral to the therapeutic environment. Incorporating this perspective into the theoretical framework allows for a more nuanced understanding of how volunteers contribute to service accessibility within youth mental health settings [18,19]. This study contributes to this underexplored area by examining how staff perceive and navigate these relational dynamics with volunteers within a youth mental health context.

### 1.3. Background

The mental health service at the centre of this study provides community, centre-based mental health care for young people aged 12 to 25 years in Melbourne, Victoria. The main purpose of the volunteer initiative was to enhance access to and the engagement of vulnerable young people seeking support for their mental health. To be eligible for the volunteer programme, individuals must be of a similar age to service users and undertaking university study in allied health fields (such as psychology, social work or occupational therapy). To become a volunteer, applicants must complete an interview and face-to-face training in ethics and care within youth mental health.

The centre was designed as a youth-friendly space which aims to support wellbeing. This is achieved through the provision of a casual space where the volunteers work, beyond the reception and waiting area, which includes a small kitchen (to make drinks), a large table, easy chairs, a bookcase with access to a book exchange, a whiteboard, access to a free computer with WIFI and various games and puzzles. The additional space can be enclosed, if needed, for privacy. The volunteers described in this study use this space to engage with the young people entering the service. This engagement involves various activities such as orienting them to the service, assisting them to complete the paperwork (via an iPad) on arrival, making a coffee or other drink or chatting. In essence the volunteers work with staff acting as a liaison between the reception staff to welcome the young person and offer a warm handover to clinical staff at the appointment time. In the event the volunteer encounters any challenging situation there are staff on hand to provide on-the-spot support. It should be noted that the inclusion of young volunteers in a mental health setting is not commonplace in youth mental health services.

The aim of this study was to explore staff perspectives on the volunteer model and its impact on service delivery. This was guided by the following research questions:

1. What are the staff experiences of the volunteer initiative?
2. How does the volunteer initiative improve engagement and access for young people?
3. What are the benefits and challenges to the implementation of a volunteer model in a youth mental health setting?

## 2. Methods

### 2.1. Study Design

This study utilised a qualitative constructivist approach, exploring staff experiences of the volunteer programme. This approach was deemed the most appropriate since it enables researchers to gain knowledge and understanding through the lived experience of participants in their own words where there is limited prior knowledge and understanding [20,21,22]. Ethics approval was obtained from Alfred Health (project number 88/22) and Monash University (project number 41312).

### 2.2. Recruitment and Participants

Purposive sampling was undertaken to recruit potential participants. All staff members were initially contacted via email with a copy of the participant information and consent form and invited to participate. Staff responded to ‘opt in’ to participate in the one-on-one interviews at a time and day that suited them. Participants provided written and verbal consent prior to the interview. A total of eight participants were interviewed, with five being clinical staff (psychologists, social workers and occupational therapists) and three being non-clinical staff (administrative). Participants were given the opportunity to review or withdraw their interview; none chose to do so.

### 2.3. Data Collection

An interview guide was developed by the authors based on the research aims, discussion within the research team and the existing literature. The guide was tested prior to data collection by a novice researcher (AG), under the guidance of experienced postdoctoral researchers (LH and MK). Following testing, the interview questions were refined. Example questions included “Can you describe the ways you interact with the volunteers?” and “How do you find working with the current volunteers?”.

Data collection occurred over an eight-week period between March and April 2024. Semi-structured interviews, led by the first author, were conducted with staff either face-to-face or online via videoconferencing. Interviews ranged from 20 to 55 min and were recorded, transcribed verbatim and deidentified. Data was securely stored on the health service server in a password-protected folder.

### 2.4. Data Analysis

The data was analysed using the six-phase process of reflexive thematic analysis (RTA) as recommended by Braun and Clarke [20]. The RTA framework was selected for its theoretical flexibility and suitability for exploring individuals’ experiences, views and perceptions [23].

Reflexive and repetitive engagement with the dataset produced a robust data analysis, where patterns of meaning (themes) were derived directly from the data to address the research aim and questions.

To support this process, author AG used memoing to document theme development. The research team met on several additional occasions to reflect, compare and expand on their own identified themes. This ensured analytical rigour as researchers were able to identify and address their own biases by reaching an agreement on the final themes. A summary of the analysis process is displayed in Table 1.

## 3. Findings

**Figure 1:**
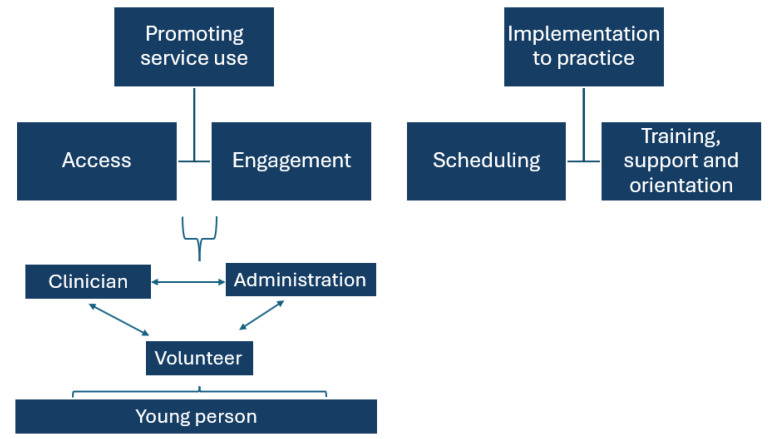
Themes and sub-themes generated from data.

Analysis of the data showed that two main themes, as illustrated in Figure 1, could be identified from the staff reports of their experiences with the volunteer programme. The first, “Promoting service use” relates to the ways in which the programme contributes to improving the experience for young people entering the service, which taps into the research question of staff experiences (RQ1) and how the volunteer initiative improved engagement and access for young people (RQ2). The second theme, “Implementation to practice”, relates to the practical implications of setting up and maintaining the programme which addressed RQ3 the benefits and challenges of the volunteer model in a youth mental health setting.

### 3.1. Promoting Service Use

The first main theme ‘promoting service use’ encompasses the ways in which staff perceived how the volunteer programme improves access and engagement for young people entering the service. The findings highlight that this was achieved through interactions and relationships between the triad of volunteers, clinicians and administrative staff. Service staff benefitted from the increased rates of completion of paperwork by and for young people, while the volunteers also benefitted through support and knowledge sharing from professionals, thereby creating a mutually beneficial cycle.

#### 3.1.1. Administrative Staff

One of the greatest benefits of the volunteers to administration staff was the assistance with the completion of paperwork, reducing workload and the associated pressure. All staff members reported that volunteers reduced pressure and workload where “volunteers sped up the process of the paperwork.” (P8) and “made [staff] workload lighter” (P7).

Administrative staff described how the large amount of paperwork was often ‘daunting and overwhelming’ for the young person, with some needing assistance to complete all the documents. However, in busy periods, juggling these requirements was challenging for administrative staff, so the volunteer involvement enabled the administration staff to “focus on other things that need our attention, instead of trying to stand there and explain what [the paperwork] is to a young person” (P5). The administration staff felt that individual assistance with the paperwork could result in the young person being “less heightened… in bit more of a calm experience and good first entry into [the centre]” (P2). This personal support can lead to the young person feeling more positive about their overall experience.

#### 3.1.2. Clinical Staff

Not only did completion of the mandatory paperwork support administration staff, it was also a benefit to clinical staff. When the paperwork was completed directly prior to the appointment, it enabled clinical staff to understand imminent and current concerns of the young person, enabling them to pivot the discussion to the area of concern. One clinician mentioned “[the paperwork provides a] heads-up on where someone might be at in that moment… it [can] guide decision making” (P4).

Ensuring the paperwork is complete prior to the appointment ensured that the clinical staff could concentrate on providing the counselling to the young person. “[The paperwork and data] helps track how well a young person is engaging and feeling listened to and understood … And that’s actually something you want to have a conversation about. If their self-reported data is indicating that it’s not feeling good for them, that’s a beautiful opportunity to try to understand what’s going on for them … that we want to try and remedy (P8).

This opening up of communication between the young person and the clinician is clearly facilitated by the youth volunteers, who can help form a bridge across the age and experience gap.

#### 3.1.3. Volunteers

Although there was a clear benefit to staff as a result of the volunteers, the participants highlighted how the volunteers also benefitted from their participation in the programme. Since the volunteers were pre-qualified students in a range of health disciplines, they were noted to benefit directly through their interactions with staff, which enabled them to connect, be a part of the community and to learn about the various roles within youth mental health.

Participants highlighted the importance of creating connections with the volunteers and how the connections occurred through casual interactions such as lunchtime conversations. Staff described volunteers as “feel[ing] like they’re part… of our little community” (P3). Staff created connections with volunteers by “inviting them to lunches… it was nice [when they joined us for lunch]. Made them feel part of the team” (P6). Connections were also made through staff emphasising that volunteers are well-supported and must be “comfortable and safe during their shift. And if they have any questions they want to bring up, it’s that open, safe space for them to do.” (P5). These connections were a clear benefit to the volunteers since they gained a deeper understanding of the mental health system as well as knowledge and advice for their future career. One participant said,

“They weren’t just having lunch with admin, they were having lunch with social workers, OTs, psychologists, and they can have that chance to ask those big questions about working in the field, make connections, work connections for future employment, possibly get mentorships exposing themselves to mental health, understanding how the system works, understanding how consent registrations in a larger organisation works.” (P6).

#### 3.1.4. The Young Person

This sub-theme highlights how staff perceived the interactions and engagement between the volunteer and the young person. This implicit and underlying theme considered the interactions between administration staff, clinical staff and volunteers as benefitting the vulnerable young person attending the service for their mental health. Participants described how the service was enhanced: “I do think it’s more welcoming… it’s a strong visual presence of …we want you here. We’re youth friendly” (P8). This appeared to be of particular importance when staff were time-poor since it meant each young person was individually greeted which helped the young person feel “more comfortable” and “safe and secure”. It was also noticed that the volunteer’s presence in the waiting room provided the young person with company instead of “…sitting on their own in a quiet waiting room” (P6). This was particularly beneficial for “people using the service for the first time, they found it very… helpful …for the anxiety” (P2).

The volunteers, being of similar age to the young person, facilitated a “more inclusive and engaging” (P1) environment for the young person. One participant reported that giving a young person the option to choose from various activities whilst they wait makes it “[less] intimidating [and more] welcoming” (P8). Staff observations and experiences indicated social connection, and enjoyable interactions were evident between the volunteer and young person. The personal characteristics of the volunteers enabled such connections to be built. One participant noted “[it] was really nice to see that a young person clearly resonated really well with a volunteer and felt like it became more of a social thing as well.” (P3).

### 3.2. Implementation to Practice

The second main theme was implementation to practice, which unpacks some of the logistics that need to be considered when running a volunteer programme, such as scheduling, rostering, training and supervision of volunteers. In particular this theme explores the benefits and challenges of this type of model of care.

#### 3.2.1. Scheduling of Volunteers

Several organisational challenges were identified around the scheduling and rostering of volunteers. There were days when numerous young people either cancelled their appointment, failed to attend or converted to telehealth at the last minute. As a result, there were fewer young people attending in person, which resulted in the volunteers having periods of downtime. One participant commented,

“On a day [when] we don’t have any young people, there’s a big gap … sometimes there was a bit of sitting around… they filled their time and they were happy to do study, but it would be great if we could have [them] on a day that [is] busier, or they can get more experience or different types [of interactions]” (P6)

Extended periods of downtime often resulted in volunteers completing various other tasks around the centre such as, wrapping the lucky dip prizes, updating and drawing on the whiteboard and adding their own ideas to the waiting room, e.g., creating a suggestion box. Such challenges sometimes resulted in limited clarity and understanding of the role and scope of volunteers, “[the volunteers are] always really happy to help with whatever… [however], I’m not sure if that’s within the role” (P3). Furthermore, participants indicated there are further improvements that could be made to scheduling, rostering and the role of the volunteer. Recommendations for volunteers included “add[ing] to the role… to fill up that gap [of spare times]” (P6) or “utilising them in more ways than just [in] the centre” (P7).

Other challenges around rostering volunteers were identified, such when staff were “really busy and… wish[ed] we had a volunteer but there was none available” (P3), and other occasions where “there were two volunteers at once… [which was] probably over doing it…” (P8). Participants also discussed working with a young student workforce. One participant reported that “it can be tough managing and working with young people because their life is so dynamic” (P7). Other participants identified the “longevity of volunteers” (P3) in that volunteers’ interest would “peak… at particular times during their studies… [but] as time marches on… they go onto the next step in their study” (P8). Additionally, there were challenges around effective communication with volunteers, since it was sometimes “hard to get them to respond to emails or come to shifts” (P5).

#### 3.2.2. Training, Supervision and Orientation

Participants indicated that training, supervision and orientation were available to upskill and support the volunteers. Prior to the commencement of their role, orientation and onboarding was provided, consisting of one “intensive training” session which gave them insight into the youth mental health service, the volunteer role and common scenarios that they would encounter. Despite volunteers being trained, participants mentioned instances where volunteers required further knowledge and skills in some scenarios; for example, when “(a) parent comes… slightly agitated or… very assertive or wanting something that… [we] might not be able to offer and not understanding that” (P2).

In order to address any gaps in knowledge or skills, clinical and administration staff initially provided supervision sessions for the volunteers, giving them a space to “talk about what’s going on and any updates” (P2) as well as seek and “provide feedback” (P6). Supervision sessions enabled volunteers to “talk through interactions they’d had with young people or families and to share their experiences and talk through any hiccups that had popped up along the way” (P4). However, participants mentioned how the supervision sessions could be inconsistent. Initially supervision was scheduled for “every three months” but due to limited demand and poor turnout, supervision was eventually discontinued. Participants attributed these challenges to “finding an appropriate time for majority of [volunteers’ availabilities]” (P7) and/or volunteers’ lack of “need” and “demand” for supervision.

### 3.3. Engaging Volunteers in Youth Mental Health

The participants in the current study were unanimously positive about the inclusion of volunteers within their centre, and benefits that the programme has for the service, the young people and the volunteers themselves. However, the findings suggest that training is a key component of such a programme to ensure the young volunteers were equipped with adequate skills in managing both the young person entering the service but also the parents. Although the participants described how the volunteers became an integrated part of the team it was clear this could be improved through including volunteers in additional centre-based activities such as morning ‘stand-ups’ and monthly meetings. Since the volunteers were primarily allied health students, it was suggested that a regular “end of the month [chat about] highlights or… feedback [on] what they benefited from the experience… what worked, what didn’t work. … It would be great for the rest of the team to see what they think” (P6). The inclusion of these changes would not only benefit the volunteers and improve their skills but would benefit both clinical and administration staff.

## 4. Discussion

The aim of the study was to understand the staff experiences of a volunteer initiative within a youth mental health service in Melbourne, Victoria, to explore what the volunteer programme adds to the service in terms of improving access and engagement for young people, as well as the implications of implementing such programmes. The concept of young volunteers in a youth mental health setting is not a regular feature in Australia, and to the authors knowledge, has not previously been reported on. As such this study provides a new and unique opportunity for youth mental health services to consider the inclusion of a volunteer programme.

The findings highlight the mutual benefits to staff, both clinical and administrative, and to volunteers. The strength of these relationship and support within the centre resulted in reduced stress and greater efficiency in managing the workload for staff. Participants felt that through easing access into the service, the young people were more engaged. In order to implement the volunteer initiative into the service there was a need to ensure adequate orientation, support and training as well as effective scheduling of the volunteers, although at times this proved to be challenging.

In line with the findings of prior research, this study identified that having volunteers assist with administrative tasks saves time and costs, enabling staff to focus on clinical tasks, reducing their administration workload and improving the quality of patient care [24]. In addition, the prior literature found that volunteers provide social and emotional support to patients and their families that those working within health care often do not have the time to provide [8,15,25]. One of the main benefits in the current model was the completion of paperwork and the obtaining of vital information to support the staff who could then support the young person more effectively. This is a common theme described in volunteer research whereby interactions between volunteer and patient can facilitate better understanding and communication between the patient and health care professionals [9,15,24].

In the current study it was evident that the volunteers created a less clinical and more welcoming environment, for both young people and their families, which reduced stress, promoted conversation and provided companionship. We posit that these qualities may result in better engagement of young people with the service, the staff and the volunteers. This finding is similar to other volunteer studies which have found that volunteers can have a positive impact on the wellbeing of patients [26].

Demand for youth mental health services in Australia and around the world continues to grow, making it imperative that young people have easy access to engaging, youth-friendly mental health services [27]. A youth-friendly service is essential since can be more effective in attracting young people without labelling or prematurely medicalising their concerns [28]. Given the need for such services, continual enhancement must occur to improve and promote help-seeking behaviour amongst young people. In the current study the volunteers interacted with those entering the service in the waiting room and the additional space provided. The waiting room is considered to be part of the therapeutic milieu and can have a significant influence on the quality of care received [29]. A supportive, engaging environment can enhance a young person’s wellbeing and recovery, creating a comfortable atmosphere that encourages engagement during therapy sessions [28,30]. This is particularly important for young people, as individuals find clinical waiting rooms daunting and stressful, a common barrier to seeking help, especially for those seeking help for the first time [29]. Additionally, a positive therapeutic environment enhances the overall user experience and positively impacts a young person’s perception of mental health services, increasing their willingness to seek help and return to the service [31]. The main finding of this study shows that volunteers can improve the way a service operates for both clinical and administrative staff. Therefore, we posit that this has a beneficial flow on effect to the young person through reduced anxiety, increased completion of the paperwork and an overall more youth-friendly and appealing environment.

### Implications for Practice

There are some key implications around the inclusion of volunteers in youth-friendly mental health services. One of the main challenges was that of managing the volunteers’ time. While the volunteers were able to remove tasks from staff to free up staff time for other duties there were also periods of downtime for the volunteers. In the current study this was managed through engaging volunteers in other tasks within the centre such as updating white board information; however, the volunteers could also be encouraged to use the space as a place to undertake their own study or enjoy some self-care strategies. The use of a dedicated part-time coordinator role for volunteers might be a viable option in a larger, busier service where there is a greater volume of clients. A further factor to consider in practice is the implementation of group clinical supervision for the volunteers. In this service in this study, supervision meetings were offered but not well attended. It is possible that the nature of a formal supervision structure was not useful to the volunteers, resulting in low attendance. Other alternative means of offering support to volunteers which do not impact their time further should be considered. For example, the current study found there were benefits to both staff and volunteers through informal interaction such as at lunchtime, and this may be one way to provide alternate support to volunteers. This social nature within the staff and volunteer role has not previously been reported on in the literature, to the authors knowledge, and is worthy of further investigation.

## 5. Limitations and Future Research

The main limitation of the study was the inclusion of staff only. For a more robust and holistic understanding, future research should consider interviews with both volunteers and young people within the youth mental health setting. Not only will this inclusion provide further triangulation of the data it will enable a greater understanding of the experience of those directly involved. The volunteer view will be reported in a forthcoming paper. Another limitation is the inconsistency in staff experiences around volunteer interactions. Participants had varying degrees of interaction with volunteers, which may have altered their perception or understanding of the volunteers. Future research should consider the timing of data collection to coincide with a particular cohort of volunteers.

## 6. Conclusions

The findings of this study suggest that a volunteer model of care can enhance the relational dynamics between service staff, clinicians and young people. This approach can improve access and engagement to mental health services ensuring that vulnerable young people receive the mental health care they need. While the role of volunteers in bridging the gap between service users and service providers has been explored elsewhere, our study has thrown light on the importance of the volunteer role in supporting administrative staff to improve access to and engagement with mental health care. In addition, an unanticipated finding highlights how services can support young future allied health professionals by providing skills, knowledge and experience that they may not easily gain elsewhere. This study has highlighted the benefits of a scalable and inexpensive volunteer model of care providing a warm, welcoming, stress-free environment for young people seeking help for their mental health. However, services need to ensure they address operational challenges such as time management and volunteer coordination prior to the implementation of a volunteer model in order to be sustainable. Improving mental health service use is critical to addressing the growing mental health crisis throughout the world. As such, the integration of volunteers in youth mental health has the potential to transform service provision enhancing access, reducing staff workload and fostering more meaningful engagement of young people.

## Figures and Tables

**Table 1:** The analysis process.

Analysis Phase	Description of Phase	Example
Familiarisation with data.	The first author (AG) used approved AI software to transcribe the recorded interviews verbatim. Subsequently AG reviewed all transcripts iteratively, noting down preliminary ideas and emergent themes.	
2. Data coding.	The first two authors (AG, MK) re-read and coded transcripts line by line. Relevant data was colour-coded into preliminary themes, organised via mind mapping, and structured into a thematic table. Then AG and MK reviewed and compared the derived codes.	Initial quote: “I think it just creates more of a community feel here rather than, you know, a clinical. Which we are. Yeah, we are a clinical service. We operate as a clinical service, but it just adds a bit more warmth, I think.” Initial code: Benefit Subtheme: Service/Centre environment.
3. Initial theme generalisation.	Through discussion between AG and MK, the thematic table was further analysed and themes that supported the interview questions were colour coded.	Refined coding for the above quote: what volunteers add to the service/centre.
4. Theme development and review.	Prior identified themes were cross-checked against the thematic tables. AG and MK explained the coding rationale to KP and LH, who independently reviewed the themes and provided feedback. Revisions were discussed, and codes/themes were modified accordingly.	Supporting secondary quote for above coding: “I think around some vibrancy and a welcoming kind of space for young people and families. Yeah. Just to make it seem like a not intimidating, welcoming space.”
5. Theme refining, defining and naming.	Themes and subthemes were refined through ongoing analysis, writing and discussion among authors. Informative theme names and clear definitions were developed to reflect the narrative.	Refined themes: promoting service use. Example subthemes: Access; Engagement. Description: Access and engagement with the services were enhanced through the volunteer and staff relationships (clinical and administration). Not only did the volunteers reduce the workload for staff, they acted as a bridge between the young person and clinician. This theme highlights how the use of volunteers can add value to the service.
6. Writing up.	AG drafted the findings and included illustrative participant quotes. Themes and sub-themes evolved through multiple iterations of writings and author discussions. All authors contributed to the final edit of results.	Example quote: “more of a community feel here rather than… clinical.”

## Data Availability

The original contributions presented in this study are included in the article. Further inquiries can be directed to the corresponding author.
